# Supplementary material for: WordCommentsAnalyzer: A windows software tool for qualitative research
Source: F1000Res. 2018 Sep 4;7:536. Originally published 2018 May 3. [Version 2] doi: 10.12688/f1000research.14819.2 (PMC6137411; doi:10.12688/f1000research.14819.2)
Supplement: Supplementary file 3 [file f1000research-7-17614-s0002.tgz › b2a79496-4ada-47c4-9682-a99ce867836d.zip!/Eight Iranian Health Journals/Archives of Rehabilitation_9.docx]

© Archives of Rehabilitation (RJ). University of Social Welfare and Rehabilitation Sciences, Koodakyar Alley, Daneshjoo Blv., Evin, Tehran, Iran. This work is licensed under [CC BY-NC 4.0](https://creativecommons.org/licenses/by-nc/4.0/).

Volume & Issue: دوره 17 - شماره 1

Title: اثربخشی مداخله زودهنگام مبتنی‌بر والدین بر میزان توجه اشتراکی (پاسخ‌دهی) کودکان پیش‌دبستانی دارای اختلال طیف اوتیسم: پژوهش مورد منفرد

http://rehabilitationj.uswr.ac.ir/article-1-1736-fa.html

هدف توجه اشتراکی، به توانایی فرد در تمرکز بر اطلاعات موجود در کانون توجه دیداری خویش، به‌موازات اطلاعات موجود در کانون توجه دیداری فرد دیگر اشاره دارد. در این جریان، دو نفر فعالانه توجه‌ خود را معطوف یک شی‌ء، موقعیت یا رویداد می‌کنند. نقص در توجه اشتراکی یکی از اولین نشانه‌های قابل‌تشخیص در کودکان دارای اختلال طیف اوتیسم به شمار می‌رود. این مهارت تأثیر بسیاری بر رشد و تحول مهارت‌های زبانی، شناختی و اجتماعی دارد. به‌عبارت‌دیگر، توجه اشتراکی ممکن است به‌عنوان یک مهارت کلیدی عمل کند؛ بدین معنی که دستیابی به این حوزه از رشد، دستاوردهای جانبی بسیاری را برای کودک به ارمغان می‌آورد. ازاین‌رو، استفاده از مداخلات درمانی زودهنگام به‌منظور بهبود توجه اشتراکی کودکان دارای اختلال طیف اوتیسم ضرورت می‌یابد. پژوهش حاضر به‌منظور بررسی اثربخشی مداخله زودهنگام مبتنی‌بر والدین بر میزان توجه اشتراکی (پاسخ‌دهی) کودکان پیش‌دبستانی دارای اختلال طیف اوتیسم انجام گرفت. روش بررسی جامعه آماری این پژوهش را کودکان مراجعه‌کننده به مرکز اوتیسم اردیبهشت در شهر اصفهان تشکیل می‌دادند که تعداد آنها 57 نفر بود. ازآنجاکه پژوهش حاضر به‌دنبال موارد خاص یا غیرمعمول بود، از روش نمونه‌گیری هدفمند استفاده شد و شرکت‌کنندگان براساس قضاوت پژوهشگر یا اهداف مطالعه انتخاب شدند. براین‌اساس، سه کودک 5-3 ساله که به تشخیص متخصص، علایم و نشانه‌های اختلال طیف اوتیسم را داشتند و در زمینه توجه اشتراکی دچار آسیب بودند، انتخاب شدند. ابزار پژوهش، مقیاس تشخیص اوتیسم گیلیام (GARS) و مقیاس ارتباط اجتماعی اولیه (ESCS) بود. در این پژوهش، روش مورد منفرد با طرح A-B مورداستفاده قرار گرفت. در این روش پس از 5 جلسه خط پایه، مداخله آغاز گردید و طی 12 جلسه انفرادی مهارت توجه اشتراکی به کودکان آموزش داده شد. به‌منظور تجزیه و تحلیل داده‌های گردآوری‌شده، ابتدا نمرات خام به نمرات استاندارد تی تبدیل و موقعیت خط پایه، مداخله و پیگیری هر آزمودنی روی نمودار رسم و برای تفسیر و نتیجه‌گیری از این نمودارها، از تحلیل دیداری، شاخص روند و شاخص ثبات، درصد داده‌های غیرهمپوش و درصد داده‌های همپوش استفاده شد. یافته‌ها در تحلیل دیداری نمودار داده‌ها، مداخله در هر سه آزمودنی اثربخش بوده است. میانگین نمرات از 92/39، 30/37 و 89/38 برای هر سه آزمودنی در خط پایه به 04/52، 79/52 و 69/51 در موقعیت مداخله رسید. درصد غیرهمپوشی داده‌ها (PND) در دو موقعیت خط پایه و مداخله  برای سه شرکت‌کننده به‌ترتیب 83، 100 و 91  درصد بود. این اثربخشی در مرحله پیگیری، همچنان مشهود بود. نتیجه‌گیری نتایج پژوهش حاضر نشان داد مداخله زودهنگام مبتنی‌بر والدین می‌تواند به‌عنوان یک روش آموزشی مؤثر برای بهبود توجه اشتراکی در کودکان پیش‌دبستانی دارای اختلال طیف اوتیسم مورداستفاده قرار گیرد. با توجه به اینکه آسیب در توجه اشتراکی، یکی از ویژگی‎‌های اصلی کودکان دارای اختلال طیف اوتیسم است و توجه اشتراکی سبب بهبود در دیگر حیطه‌های رشدی می‌شود، می‌توان پیشنهاد کرد که بهبود توجه اشتراکی باید به‌عنوان یک هدف اصلی در مداخلات توان‌بخشی و آموزشی در کودکان دارای اختلال طیف اوتیسم تعیین شود.

Title: بررسی تأثیر کفی دارای بافت برجسته به صورت یک طرفه بر تقارن تحمل وزن و پارامترهای راه رفتن در بیماران سکته مغزی مزمن

http://rehabilitationj.uswr.ac.ir/article-1-1780-fa.html

هدف عدم‌تقارن در تحمل وزن یکی از علت‌های اصلی ایجاد اختلال در حفظ تعادل بیماران همی‌پارزی است که می‌تواند ایستادن و راه‌رفتن این بیماران را با مشکل مواجه کند. این مطالعه با هدف بررسی تأثیر آنی کفی دارای بافت برجسته به‌صورت یک‌طرفه بر تقارن تحمل وزن در حالت ایستاده و راه‌رفتن افراد سکته مغزی مزمن انجام شد. روش بررسی در این مطالعه شبه‌تجربی، 16 بیمار همی‌پارزی با میانگین سنی 94/6±12/52 سال و متوسط زمان گذشته از ضایعه 4/16±12/33 ماه با معیارهای گذشت حداقل 6 ماه از ضایعه، تجربه اولین سکته مغزی براساس تشخیص پزشک، توانایی راه‌رفتن حداقل 10 متر و ایستادن حداقل 30 ثانیه بدون وسیله کمکی، اسپاستی‌سیتی کمتر از 2 براساس معیار آشورث تصحیح‌شده در اندام تحتانی و همچنین نداشتن هیچ‌گونه مشکل ارتوپدی و نورولوژیکی دیگر، به‌صورت غیراحتمالی ساده انتخاب شدند. شاخص تقارن در حالت ایستاده (با استفاده از دو ترازو مشابه)، قرینگی طول گام، طول گام و سرعت راه‌رفتن (با استفاده از دستگاه بالانس مستر نوروکام) در 3 حالت بدون کفی، کفی دارای بافت برجسته با سختی 80 و کفی دارای بافت برجسته با سختی 60 محاسبه شد. یافته‌ها در این مطالعه برای مقایسه سه حالت آزمون از آزمون آماری تحلیل واریانس در تکرار مشاهدات و برای مقایسه دو‌به‌دو از آزمون بونفرونی استفاده شد. قرینگی طول گام تفاوت معنی‌داری بین حالت بدون کفی و حالت استفاده از کفی دارای بافت برجسته با سختی 80 (004/0=P) و همچنین استفاده از کفی دارای بافت برجسته با سختی 80 در مقایسه با کفی دارای بافت برجسته با سختی 60 (011/0=P) نشان داد. باوجوداین، در حالت استفاده از کفی دارای بافت برجسته با سختی 60 در مقایسه با حالت بدون کفی، تفاوت معنی‌داری مشاهده نشد (325/0=P). نتایج شاخص تقارن مانند قرینگی گام بود. بین نتایج حالت بدون کفی در مقایسه با استفاده از کفی دارای بافت برجسته با سختی80 (022/0=P) و بین نتایج دو نوع کفی (019/0=P) تفاوت معنی‌دار بود، اما در استفاده از کفی دارای بافت برجسته با سختی 60 نسبت به حالت بدون کفی با وجود بهبود قرینگی طول گام، نتایج معنی‌دار نشد (325/0=P). سرعت راه‌رفتن و طول گام در هنگام استفاده از هیچ‌یک از کفی‌ها به‌طور معنی‌دار بهبود نیافت. نتیجه‌گیری مطالعه حاضر نشان داد که استفاده اجباری از اندام سمت مبتلا قادر است با غلبه بر پدیده عدم‌استفاده آموخته‌شده و اصلاح عارضه نقص در ارسال پیام‌های حسی به مراکز کنترل‌کننده حرکت، باعث بهبود تقارن تحمل وزن در حالت ایستاده و راه‌رفتن بیماران سکته مغزی مزمن شود. براساس نتایج این مطالعه، استفاده از کفی دارای بافت برجسته با سختی 80 به‌صورت یک‌طرفه تقارن تحمل وزن در حالت ایستاده و قرینگی طول گام در افراد همی‌پارزی را بهبود می‌بخشد، ولی بر سرعت راه‌رفتن و طول گام این افراد تأثیری ندارد. کفی دارای بافت برجسته با سختی 60 هیچ‌یک از متغیر آزمون را به‌صورت معناداری بهبود نبخشید. برهمین‌اساس، از این نوع کفی‌ها می‌توان در تمرینات تعادلی و راه‌رفتن افراد همی‌پارزی استفاده کرد.

Title: بررسی تأثیر لبه خارجی و قوس طولی داخلی بر جا‌به‌جایی نیروی عکس‌العمل زمین در بیماران مبتلا به استئوآرتریت زانو

http://rehabilitationj.uswr.ac.ir/article-1-1752-fa.html

هدف استئوآرتریت نوعی بیماری تخریبی مفاصل است که در آن سطوح مفصلی دچار فرسایش و خوردگی می‌شوند. به‌دنبال ایجاد استئوآرتریت، حرکات یکنواخت و روان مفصل دچار اختلال می‌شود. استئوآرتریت شایع‌ترین نوع آرتریت است که شیوع این بیماری در ایران 3/19 درصد است و افراد مبتلا به استئوآرتریت زانو معمولاً دچار ژنوواروم و هایپر پرونیشن پا هستند. هدف این مطالعه بررسی جابه‌جایی نیروی عکس‌العمل زمین نسبت به زانو با توجه به پاسخ ایستا زانو به لبه خارجی و قوس داخلی و ترکیب قوس با لبه خارجی بود. روش بررسی این پژوهش، نیمه‌تجربی بود و نمونه‌گیری از جامعه به روش نمونه‌گیری دردسترس شامل 13 نفر افراد مبتلا به استئوآرتریت زانو 9 زن و 4 مرد با میانگین سنی 7/7±08/63 انجام گرفت. در پژوهش حاضر چهار حالت آزمون بدون کفی، لبه خارجی، قوس داخلی و ترکیب قوس با لبه خارجی موردآزمایش قرار گرفت. برای اندازه‌گیری تغییرات جابه‌جایی نیروی عکس‌العمل زمین، از دستگاه لیزر پاسچر و سنجش میزان راحتی پرسشنامه VAS استفاده و تغییرات بازوی اهرمی با دستگاه اندازه‌گیری شد. داده‌ها با استفاده از نسخه 20 نرم‌افزار SPSS و آزمون چندمتغیره و سپس دو‌به‌دو بونفرونی مورد تجزیه و تحلیل قرار گرفت. یافته‌ها لبه خارجی باعث کاهش معنادار (005/0P=) فاصله نیروی عکس‌العمل زمین تا مرکز زانو نسبت به حالت بدون کفی می‌شود. میانگین فاصله خط وزن از مرکز زانو در حالت بدون ارتوز 6/13میلیمتربه داخل، با لبه خارجی 8/0میلیمتربه داخل، با قوس داخلی 8/11میلیمتربه داخل و با لبه خارجی با قوس داخلی 7میلیمتربه داخل بدست آمد. بنابراین استفاده از لبه خارجی به ضخامت 7 میلی‌متر باعث جابه‌جایی محل عبور نیروی عکس‌العمل زمین روی زانو شده است، به‌طوری‌که بازوی اهرمی نیروی عکس‌العمل زمین از مرکز زانو -که باعث تولید گشتاور ادداکتوری روی زانو می‌شود-، با گذاشتن لبه خارجی 8/12 میلی‌متر کوچک‌تر از وضعیت بدون کفی شده بود. در مقایسه بین دیگر حالت‌ها -یعنی بدون کفی-، ترکیب قوس داخلی با لبه خارجی و قوس داخلی به‌تنهایی، اختلاف معناداری مشاهده نشده است، اما اختلاف معناداری بین لبه خارجی و قوس داخلی به‌تنهایی به‌دست آمد (013/0=P)؛ به این صورت که بازوی اهرمی نیروی عکس‌العمل زمین با لبه خارجی 11 میلی‌متر کوچک‌تر است و همچنین در مقایسه لبه خارجی و ترکیب قوس با لبه خارجی نشان داد که لبه خارجی 2/6 میلی‌متر بازوی اهرمی کوتاه‌تر یا 5/88 درصد بازوی اهرمی کوچک‌تری ایجاد می‌کند (001/0=P). در مقایسه بین حالت‌های قوس داخلی و ترکیب قوس با لبه خارجی رابطه معناداری ملاحظه نشد.  نتیجه‌گیری لبه خارجی باعث کاهش بازوی اهرمی نیروی عکس‌العمل زمین نسبت به زانو در بیماران مبتلا به استئوآرتریت زانو با ژنووروم و هایپر پرونشن پا می‌شود و افزودن قوس طولی باعث کاهش اثر لبه خارجی برای کاهش بازوی اهرمی نیروی عکس‌العمل زمین نسبت به زانو و درنتیجه، عدم‌کاهش گشتاور ادداکتوری زانو در بیماران مبتلا به استئوارتریت زانو می‌شود. بااین‌حال از نظر آماری لبه خارجی، قوس داخلی و ترکیب آنها تأثیری بر راحتی در این بیماران ندارند.

Title: مطالعه موردی: تأثیر تمرینات مقاومتی پیشرونده و تعادلی بر قدرت عضلانی بالاتنه کودکان فلج مغزی

http://rehabilitationj.uswr.ac.ir/article-1-1790-fa.html

هدف فلج مغزی، به آسیب مغزی غیرپیشرونده ناشی از تکامل غیرطبیعی مغز یا صدمات مغزی در پیش، حین و مدتی پس از تولد گفته می‌شود. دلیل ایجاد فلج مغزی، صدمه مغزی یا غیرطبیعی بودن مغز است و بیشتر این معضل‌ها هنگامی‌که کودک در شکم مادر قرار دارد، اتفاق می‌افتد ولی احتمال وقوع آنها در دو سال اول زندگی -یعنی زمانی‌که مغز در حال تشکیل است- نیز زیاد است. یکی از شایع‌ترین و مهم‌ترین علامت ناتوان‌کننده فلج مغزی، آسیب به عملکرد فوقانی است، به‌طوری‌که کنترل حرکات در گرفتن و رهاکردن با مشکل مواجه و درنهایت، سبب کاهش قدرت دست برنامه‌ریزی حرکتی می‌شود. مطالعات مختلفی تأثیر تمرینات مقاومتی بر قدرت عضلات افراد فلج مغزی (CP) را بررسی کرده‌­اند، درحالی‌که پژوهشی درباره تأثیر تمرینات مقاومتی پیشرونده و تعادلی در کودکان فلج مغزی انجام نشده است. هدف پژوهش حاضر، بررسی تأثیر تمرینات مقاومتی پیشرونده و تعادلی بر قدرت عضلات بالاتنه کودکان فلج مغزی بود. روش بررسی تعداد 3 پسر فلج مغزی (2 نفر با سن 7 سال و 1 نفر با سن 6 سال) در این طرح مطالعاتی شرکت کردند. در این پژوهش از روش پژوهشی هدفمند با طرح A-B-A استفاده شده است. تمرینات مقاومتی پیشرونده و تعادلی به‌مدت هشت هفته و سه جلسه در هفته پیگیری و قدرت عضلات خم‌کننده مچ دست و خم‌کننده‌های آرنج توسط دستگاه دینامومتر پاور تِرَک Õ ساخت شرکت جی.تِک با آستانه 4/4 نیوتن اندازه‌گیری شد. یافته‌ها یافته‌ها نشان داد براساس تحلیل دیداری نمودار داده‌ها و شاخص‌های آمار توصیفی و تحلیل دیداری، تمرینات مقاومتی و تعادلی در موقعیت مداخله نسبت به خط پایه باعث افزایش قدرت عضلات خم کننده آرنج شد (PND برای شرکت‌کننده اول و دوم 75 درصد و برای شرکت‌کننده سوم 100 درصد به‌دست آمده است). یافته‌های حاصل از تحلیل دیداری نمودار داده‌های هر سه آزمودنی نشان داد قدرت عضلات خم‌کننده مچ دست هر سه آزمودنی در موقعیت مداخله نسبت به خط پایه افزایش داشت (PND برای شرکت‌کننده اول و سوم 75 درصد و برای شرکت‌کننده دوم 100 درصد به‌دست آمد). میزان قدرت عضلات بالاتنه افراد بعد از پایان مداخله نسبت به مرحله پایه بهبود اما یک‌ماه پس از پایان مرحله مداخله، روند نسبتاً ثابتی داشته است.  نتیجه‌گیری پژوهش حاضر نشان داد تمرینات مقاومتی و تعادلی بر قدرت عضلات کودکان فلج مغزی تأثیر معناداری دارد. به‌نظر می‌رسد تمرینات انجام‌شده ویژه عضلات خم‌کننده مچ دست و آرنج بوده بنابراین می‌توان گفت افزایش قدرت عضلات کودکان فلج مغزی از راه اصل ویژگی تمرین و با افزایش سازگاری عصبی رخ داده است. یکی از نکات مهم در تأثیر تمرین مقاومتی، شدت تمرین است. نتایج نشان داد تمرینات مقاومتی و تعادلی باعث افزایش قدرت عضلات در کودکان مبتلا به فلج مغزی شده است. بخشی از افزایش قدرت می‌تواند ناشی از افزایش حجم عضلات و بخشی دیگر ناشی از هورمون‌های آنابولیکی نسبت داده شود.
